# Thermomechanical and Fire Properties of Polyethylene-Composite-Filled Ammonium Polyphosphate and Inorganic Fillers: An Evaluation of Their Modification Efficiency

**DOI:** 10.3390/polym14122501

**Published:** 2022-06-20

**Authors:** Mateusz Barczewski, Aleksander Hejna, Kamila Sałasińska, Joanna Aniśko, Adam Piasecki, Katarzyna Skórczewska, Jacek Andrzejewski

**Affiliations:** 1 Institute of Materials Technology, Faculty of Mechanical Engineering, Poznan University of Technology, Piotrowo 3, 61-138 Poznan, Poland; joanna.anisko@put.poznan.pl (J.A.); jacek.andrzejewski@put.poznan.pl (J.A.); 2 Department of Polymer Technology, Gdansk University of Technology, Narutowicza 11/12, 80-233 Gdansk, Poland; 3 Faculty of Materials Science and Engineering, Warsaw University of Technology, Wołoska 141, 02-507 Warsaw, Poland; 4 Department of Chemical, Biological and Aerosol Hazards, Central Institute for Labour Protection—National Research Institute, Czerniakowsa 16, 00-701 Warsaw, Poland; 5 Institute of Materials Engineering, Faculty of Materials Engineering and Technical Physics, Poznan University of Technology, Jana Pawła II 24, 60-965 Poznan, Poland; adam.piasecki@put.poznan.pl; 6 Faculty of Chemical Technology and Engineering, Bydgoszcz University of Science and Technology, Seminaryjna 3, 85-326 Bydgoszcz, Poland; katarzyna.skorczewska@pbs.edu.pl

**Keywords:** polyethylene, composite, fire behavior, fire retardant, copper slag, basalt powder, expanded vermiculite

## Abstract

The development of new polymer compositions characterized by a reduced environmental impact while lowering the price for applications in large-scale production requires the search for solutions based on the reduction in the polymer content in composites’ structure, as well as the use of fillers from sustainable sources. The study aimed to comprehensively evaluate introducing low-cost inorganic fillers, such as copper slag (CS), basalt powder (BP), and expanded vermiculite (VM), into the flame-retarded ammonium polyphosphate polyethylene composition (PE/APP). The addition of fillers (5–20 wt%) increased the stiffness and hardness of PE/APP, both at room and at elevated temperatures, which may increase the applicability range of the flame retardant polyethylene. The deterioration of composites’ tensile strength and impact strength induced by the presence of inorganic fillers compared to the unmodified polymer is described in detail. The addition of BP, CS, and VM with the simultaneous participation of APP with a total share of 40 wt% caused only a 3.1, 4.6, and 3 MPa decrease in the tensile strength compared to the reference value of 23 MPa found for PE. In turn, the cone calorimeter measurements allowed for the observation of a synergistic effect between APP and VM, reducing the peak heat rate release (pHRR) by 60% compared to unmodified PE. Incorporating fillers with a similar thermal stability but differing particle size distribution and shape led to additional information on their effectiveness in changing the properties of polyethylene. Critical examinations of changes in the mechanical and thermomechanical properties related to the structure analysis enabled the definition of the potential application perspectives analyzed in terms of burning behavior in a cone calorimetry test. Adding inorganic fillers derived from waste significantly reduces the flammability of composites with a matrix of thermoplastic polymers while increasing their sustainability and lowering their price without considerably reducing their mechanical properties, which allows for assigning developed materials as a replacement for flame-retarded polyethylene in large-scale non-loaded parts.

## 1. Introduction

Polyethylene (PE), despite being many decades since its first application, due to its unique properties, resulting from the possibilities for broad adjustments of its structural parameters, including its polydispersity, molecular weight, or copolymerization with other polymers, is still gaining ground in the field of scientific research [1,2,3]. The low price of this polymer, high chemical resistance, and excellent processability are the most frequently mentioned features that justify the fact that it is the most commonly processed polymer material. On the other hand, PE is a low-melting polymer characterized by high flammability. One of the thermoplastic polymers’ most frequently used modification methods is their application as a matrix in polymer composites. The scope of the research carried out and the introduction of powder or fibrous fillers included both the use of inorganic materials and plant-based fillers [4,5,6,7,8]. While quite a lot of attention is paid to the use of waste fillers of plant origin, at the same time, one should bear in mind the need to utilize inorganic compounds generated, among others, during production and technological processes in the metallurgical and mining industries. As the research [9,10] has shown, the introduction of low-processed inorganic fillers in the form of powders, in most cases, leads to the deterioration of the composites’ strength. Nevertheless, the enhancement of stiffness at elevated temperatures is noticeable, which is quite auspicious.

Much effort in the previous considerations has been devoted to increasing the temperature and thermomechanical stability and improving the fire resistance of polyethylene. Therefore, considering the high thermal resistance of inorganic fossil fillers, their use for these purposes was justified. However, it should be noted that the effects of introducing inorganic fillers, including nanometric ones, often lead to the achievement of entirely different thermal stability effects in terms of the increase mentioned above [11]. The result of the modification depends strictly on the filler’s chemical structure, size, roughness, and the specific surface area of its particles. For example, montmorillonite (MMT) often induces different effects on the polymers’ thermal stability depending on its dispersion in the matrix. The intercalated montmorillonite, in many reports, showed a negative impact on the thermal stability of the polymeric matrix, which was attributed to the presence of cationic compounds used for its modification.

On the other hand, the exfoliation of MMT yielded thermal stabilization of various polymers due to finer particles’ dispersion [11]. Many published studies have shown that adding inorganic fillers can inhibit the burning rate, reduce smoke emission, lower the heat release rate, and increase char formation [12]. Often, the impact of temperature-stable powder fillers, such as silica [13], basalt [14], or metal oxides [15], which are not reactive with multiple thermoplastic polymer matrices, is mainly related to the reduction in the amount of organic polymer constituting the fuel during the burning process. Considering the results presented by Motahari et al. [13], it should also be considered that the addition of highly porous inorganic fillers may affect the thermal conductivity of polymers, which may directly influence the change in the combustion process. 

Unfortunately, as demonstrated by several studies [12,14,16], the addition of sole fillers without flame retardants that causes additional, complex behavior during the fire, including intumescent effects, was insufficient. Recent research has shown that the effective modification of polymers is often achieved through synergy between multiple modifiers and fillers introduced into them. This phenomenon is successfully used to produce complex flame-retardant systems [17,18,19]. In the reported works developed, it has been shown that the simultaneous use of inorganic fillers with dedicated flame retardants can bring beneficial effects with regard to the overall flammability of polymer composites [17,20,21]. In such cases, increasing the efficiency of the powder fillers in the composition is not only based on reducing the amount of polymer. Additional benefits may be attributed to the barrier effects toward free radicals due to the presence of thermally stable fillers with a plate structure, such as exfoliated phyllosilicates [21]. The process of using modified mesoporous silica was also described, which led to the physical protection and hindered the volatilization of the oligomers due to the filler’s agglomeration in the composite’s surface layer during exposure to the flame [17].

Apart from the beneficial flammability reduction obtained by the simultaneous introduction of flame retardants and inorganic fillers, which is required by the most demanding industries such as the automotive industry [22], it is also essential to analyze obtained materials while considering the full spectrum of their properties, including their mechanical and thermomechanical performance. Many systems containing significant amounts of fire retardants (20–30 wt%) are not considered composites [23]. The additional introduction of powder or plate-shaped inorganic fillers with a low aspect ratio results in generating a hybrid structure containing insoluble particles with different properties and limited adhesion dispersed in the polymer matrix. Due to the insufficient interfacial interactions, the reinforcing effect on the polymer matrix is often limited. Moreover, a high share of the flame retardants required to achieve the expected flammability class intensifies the reduction in mechanical properties. Therefore, the substantive analysis of the correlation between the incorporation of additional inorganic, highly temperature-stable fillers into fire-retarded polymers is still an actual research topic. The significant deterioration of the mechanical performance is often ignored or omitted. The analysis of mechanical properties does not emphasize the limitations resulting from the total share of additives and fillers (up to 70 wt%) [24].

The study aimed to comprehensively evaluate introducing low-cost inorganic, fossil, and waste fillers into the flame-retarded polyethylene composition. Critical examinations of changes in the mechanical and thermomechanical properties related to the structure analysis enabled the definition of the potential application perspectives analyzed in terms of burning behavior in a cone calorimetry test. Incorporating three fillers with similar thermal stability but differing particle size distributions and shapes yielded additional information on their effectiveness in changing the properties of polyethylene.

## 2. Experimental

### 2.1. Materials

The commercial-injection-molding grade of high-density polyethylene (HDPE), type M300054, delivered by SABIC (Netherlands), was applied as a matrix for preparing composites. According to producer data, its density is 0.954 g/cm^3^, and it has a melt flow rate (MFR) of 30 g/10 min (190 °C, 2.16 kg). 

The intumescent flame retardant used for modification of the polyethylene was commercial Exolit AP 422, delivered by Clariant. It is a composition based on an ammonium polyphosphate (APP). 

Three different inorganic fillers were used for manufacturing the composites: copper slag (CS), basalt powder (BP), and expanded vermiculite (VM). Copper slag (CS), a by-product generated from a suspension furnace, was derived in the form of fine powder with a grain density of 3.04 g/cm^3^ from Polish copper-rich deposits. The chemical composition of CS, as declared by the supplier, consists of: 41.2 wt% SiO_2_, 19.1 wt% Al_2_O_3_, 13.1 wt% CaO, 12.0 wt% Fe_2_O_3_ + FeO, 4.9 wt% MgO, 1.1 wt% Cu. The material showed a moisture content of 0.13 wt%. Natural BP, with a density of 2.95 g/cm^3^, was a waste product obtained from the production of asphalt aggregate in Poland. Dominant chemical ingredients of BP given by the supplier in the technical data sheets were: 47.89 wt% SiO_2_, 15.17 wt% Al_2_O_3_, 10.92 wt% Fe_2_O_3_, 9.49 wt% CaO, 7.57 wt% MgO, 3.33 wt% Na_2_O, 2.04 wt% TiO_2_, 0.90 wt% K_2_O, 0.55 wt% P_2_O_5_, 0.20 wt% MnO, 0.01 wt% SO_3_, and 0.01 wt% F. Thermally expanded vermiculite (VM) before pre-processing was characterized with a density of 2.61 g/cm^3^ and a particle size up to 1.6 mm. It was provided by Perlit Polska (Poland). Before use, VM was subjected to milling with the knife mill Retsch GM200 with a knife rotational speed of 5000 rpm and 5 min, and was sieved by a Fritsch Analysette 3 mechanical siever using 100 µm mesh. The annealing process was carried out at a temperature of 1260 °C, and the chemical composition according to the manufacturer’s data is 38.0–49.0% SiO_2_, 20.0–23.5% MgO, 12.0–17.5% Al_2_O_3_, 0.3–5.4% Fe_2_O_3_, 5.2–7.9% K_2_O, 0.0–1.2% FeO, 0.7–1.5% CaO, 0.0–0.8% Na_2_O, 0.0–1.5% TiO_2_, 0.0–0.5 Cr_2_O_3_, 0.1–0.3% MnO, 0.0–0.6% Cl, 0.0–0.6% CO_2_, 0.0–0.2% S. Broader information about the used fillers was presented in a previous works [25,26,27].

### 2.2. Sample Preparation

The composites were prepared by mixing them in a molten state. The HDPE pellets were pulverized into a fine powder using a Tria 25-16/TC-SL high-speed knife grinder to facilitate a more efficient physical mixing process with powdered organic filler. The polymeric powder was then preliminary mixed with 20 wt% of APP and 5, 10, and 20 wt% of filler using a Retsch GM200 knife mixer (5 min, 3000 rpm). Before being mixed in the molten state, the compositions were dried in a laboratory cabined dryer Memmert ULE 500 for 12 h at 70 °C. The mixtures were processed using a ZAMAK EH16.2D co-rotating twin-screw extruder operating at 100 rpm, with a maximum temperature for the process of 190 °C. For tensile and impact strength tests, the specimens with dimensions 100 × 100 × 4 mm^3^ were manufactured with an Engel HS 80/20 HLS injection molding machine operating at 210 °C. The injection molding process was conducted with the following parameters: mold temperature T_mold_ = 30 °C, injection speed V = 100 mm/s, forming pressure P_f_ = 5 MPa, and cooling time t = 60 s. Standardized specimens for mechanical testing were mechanically processed.

### 2.3. Methods

The particle size distribution of inorganic fillers was characterized using a laser particle sizer Fritsch ANALYSETTE 22 apparatus (Weimar, Germany) operating in the range of 0.08–2000 μm.

Scanning electron microscopy (SEM) was performed using the model Tescan MIRA3 microscope (Brno-Kohoutovice, Czech Republic). The measurements were conducted with an accelerated voltage of 5 kV and magnifications of 200× and 2000×. The measurements were conducted with an accelerated voltage of 12 kV in the backscattered electrons (BSE) and secondary electron (SE) modes. The thin carbon coating (~20 nm) was deposited on samples using the Jeol JEE 4B vacuum evaporator. 

The Fourier transform infrared spectroscopy (FT-IR) measurements were realized using a spectrometer Jasco FT/IR-4600 (Tokyo, Japan) at room temperature (23 °C) in the Attenuated Total Reflectance (ATR - FT-IR) mode. A total of 32 scans at a resolution of 4 cm^−1^ were used in all cases to record the spectra. 

The specific weight of the applied fillers and resulting composites was determined using a gas pycnometer Pycnomatic from Thermo Fisher Scientific Inc. (Waltham, MA, USA). The following measurement settings were applied: gas—helium; target pressure—2.0 bar (29.0 psi); flow direction—reference first; temperature control—on; temperature set—20.0 °C; cell size—medium, 40 cm^3^; the number of cleaning cycles—3; the number of measurements—10.

The results obtained from the pycnometric measurements were used to determine the porosity of the composites as the difference between the theoretical and experimental density values. The theoretical values were calculated according to Equation (1):

(1) $$\rho_{theo}=\rho_{m}\cdot \left(1-\varphi \right)+\rho_{f}\cdot \varphi$$

where: *ρ_theo_*—theoretical density of the composite, g/cm^3^; *ρ_m_*—density of the matrix, g/cm^3^; *ρ_f_*—density of the filler, g/cm^3^; and *φ*—a volume fraction of the filler.

To quantitatively determine the composite’s porosity, Equation (2) was applied as follows:

(2) $$p=\frac{\rho_{theo}-\rho_{exp}}{\rho_{theo}}\cdot 100\%$$

where: *p*—porosity of the material, %; and *ρ_exp_*—an experimental value of composite density, g/cm^3^.

The thermal diffusivity measurements were prepared using a modified Angström method with a Maximus (Poland, Poznan) apparatus. A more comprehensive description of the experiments was described in detail in the literature [28,29]. During investigations, the microheater was charged by 23 V to heat the samples in a time of 400 s.

The thermal properties of the studied materials were analyzed using the differential scanning calorimetry (DSC) method. Samples of 5 ± 0.2 mg were placed in aluminum crucibles with pierced lids and were heated from 20 °C to 200 °C with a rate of 10 °C/min, held at this temperature for 10 min, and then cooled back to room temperature with a cooling rate of 10 °C/min. The procedure was realized twice. A Netzsch DSC 204F1 Phoenix (Selb, Germany) apparatus and an inert nitrogen atmosphere were used. The crystallinity degree *X_cr_* was calculated according to Formula (3):

(3) $$X_{cr}=\frac{\Delta H_{m}}{\left(1-f\right)\cdot \Delta H_{100\%PE}}\cdot 100\%$$

where: Δ*H_m_*—melting enthalpy of a sample, Δ*H*_100%*PE*_—melting enthalpy of 100% crystalline PE, Δ*H*_100%*PE*_ = 288 J/g [30], and *f* is the filler content.

Thermogravimetric analysis (TGA) was used to study the thermal decomposition of polyethylene and its composites. The 10 ± 0.2 mg samples were heated in the temperature range of 25–900 °C with a 10 °C/min heating rate using a Netzsch TG209 F1 (Selb, Germany) apparatus. The measurements were realized using Al_2_O_3_ crucibles in an inert atmosphere (nitrogen). The first mass derivative (DTG) was calculated in reference to the obtained mass vs. temperature curves. The 5% mass loss (T_5%_) and residual mass at 900 °C were determined.

The cone calorimeter measurements were conducted using a Fire Testing Technology Limited (UK, East Grinstead) apparatus according to the ISO 5660 standard to identify the burning behavior under forced-flaming conditions. The samples of 100 × 100 × 4 mm^3^ were placed horizontally at 25 mm below a conical heater and tested at a heat flux of 35 kW/m^2^ with piloted ignition. All samples were tested three times. The residues were photographed using an EOS 400 D digital camera from Canon Inc. (Tokyo, Japan).

The elastic modulus, elongation at break, and yield strength were tested through tensile testing. The tensile tests were performed per ISO 527 with a Zwick/Roell Z020 tensile tester model 5101 (Ulm, Germany) at room temperature. The elastic modulus measurements were conducted at a cross-head speed of 1 mm/min, while a different part of the experiment was carried out at 50 mm/min. Nine samples of each kind were tested.

The impact strength of the unnotched samples was examined by the Charpy method according to the ISO 179 standard at 25 °C. The Zwick/Roell HIT 25P (Ulm, Germany) impact tester with a 5 J hammer was applied for the measurement, and the peak load was determined as the maximum force (*Fmax*). For each series, seven specimens were tested.

The hardness was evaluated using a KB Prüftechnik (Hochdorf-Assenheim, Germany)apparatus with a ball indentation hardness test according to the ISO 2039 standard. The presented averaged values were based on a minimum of 15 tests from each series.

Vicat softening point temperature (VST) and heat deflection temperature (HDT) investigations were prepared with the use of a CEAST HV3 apparatus (Pianezza, Italy). The measurements were carried out in an oil bath following the ISO 306 standard in the A50 measurement configuration (50 N, 50 °C/h) and ISO 75 (0.455 MPa), respectively. The experiments were conducted for six specimens from each series.

## 3. Results and Discussion

### 3.1. Fillers’ Characterization

**Figure 1:**
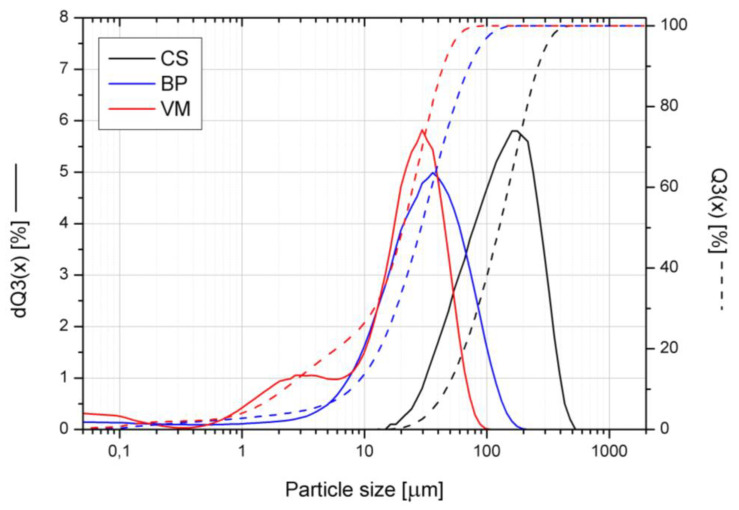
Particle size distribution of inorganic fillers.

The cumulative size distribution Q3(x) and adequate histograms dQ3(x) made for the three inorganic fillers used in this study are presented in Figure 1. The analysis of the graphs shows that the copper slag has the largest particle size, while in the case of the other fillers, most of the filler particles are of a comparable size. The VM exhibits two modes of particle size distribution due to the fraction of finely divided filler plates formed during the grinding of the expanded filler.

### 3.2. Structural Analysis

**Figure 2:**
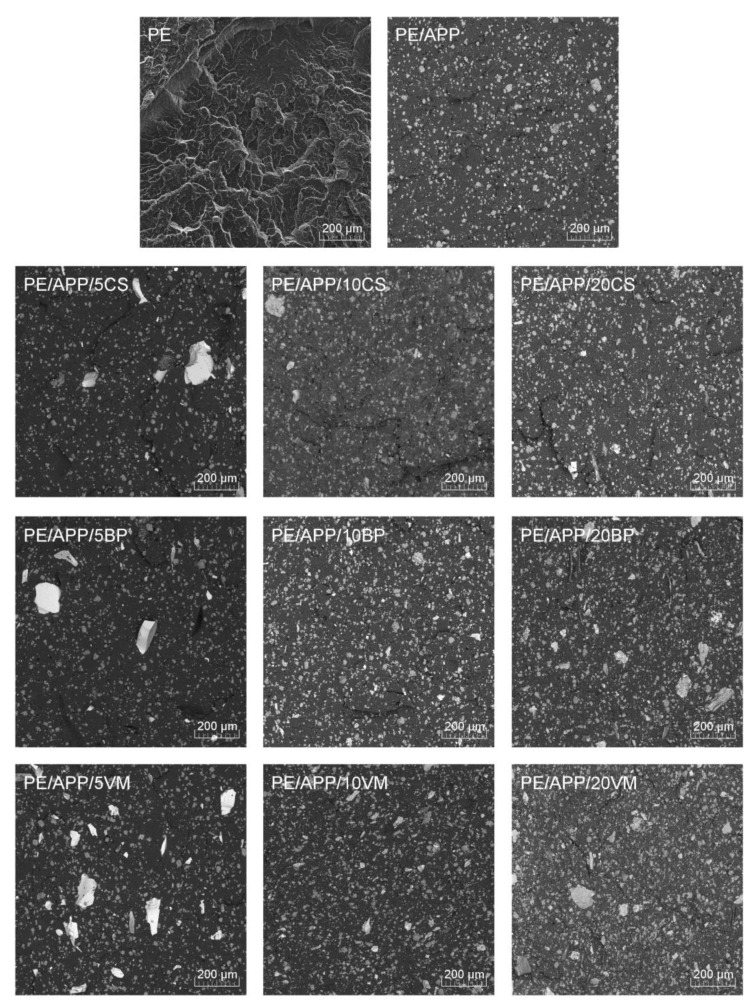
SEM images of PE and PE composites’ brittle fractures (mag. 200×, BSE mode).

**Figure 3:**
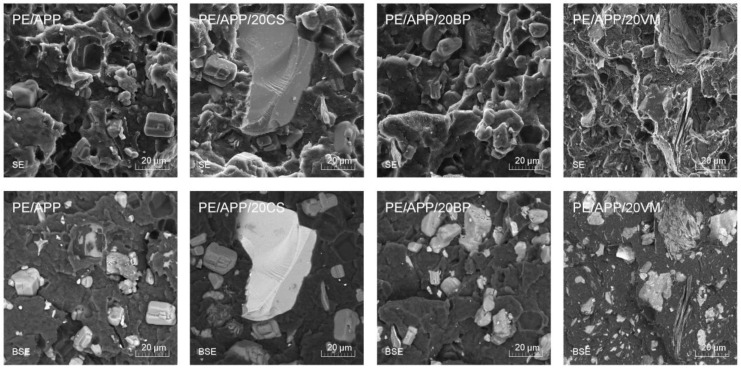
SEM images of PE and PE composites’ brittle fractures (mag. 2000×, SE and BSE mode).

Figure 2 and Figure 3 compile brittle fracture SEM images of polyethylene and its composites. Figure 2 summarizes the images taken with low magnification to evaluate the fillers’ dispersion in the polyethylene matrix. The use of the BSE mode allowed for the differentiation of the applied fillers’ particles from the APP; thanks to the various density of materials, they could be distinguished in the obtained SEM images. In the case of all materials, a homogeneous distribution of the flame retardant in the polymer matrix can be observed. In the case of composites containing BP and VM, the distribution of the filler particles also does not raise any significant concerns. It allows the compositions to be defined as homogeneous. Modified CS composites with a much larger particle size in the tested area of the series with a lower filler concentration (5 and 10 wt%) revealed the presence of larger filler fragments, while only in the case of the PE/APP/20CS composite were different sizes of copper slag particles observed to be distributed evenly on the whole analyzed area. However, based on the analysis performed, it can be concluded that none of the fillers used tended to create agglomerated structures in the PE matrix. Moreover, the introduction of inorganic fillers in various concentrations did not deteriorate the APP dispersion. Despite the long process of drying, for the composites containing in their structures natural composite inorganic BP and VM fillers, an increased number of micropores were noted, which may come from the residual moisture from the filler. 

Additionally, Figure 3 summarizes the SEM images taken in two modes, SE and BSE, for the PE/APP composition and composites, demonstrating the highest concentration of the filler (20 wt%) that can both distinguish the presence of filler particles and APP as well as assess the nature of the breakthrough. These images were taken at a higher magnification, making it possible to evaluate the adhesion at the polymer–filler interface indirectly. The break-out sites of APP particles of a regular shape are observed for all compositions. It can be concluded that the particles of inorganic fillers are characterized by better adhesion; in their case, there were no irregular pull-out holes and gaps in the interfacial region, which could suggest a loss of cohesion between the composite material and the combined materials. In the case of composites containing expanded vermiculite, broken fragments of the plate filler distributed in the sample volume are visible, as are the structures of non-comminuted filler packages that PE has not intercalated. The partial disintegration of the filler into the micrometric form of well-dispersed plates may be beneficial from the point of view of obtaining a limited flame effect [31,32]. Due to using a low processing temperature, degradation of the APP during processing may be omitted, which is also confirmed by the regular shape of the fire-retardant particles and the lack of voids at the PE/APP interface.

Table 1 summarizes the results of the physical properties measurements. Based on the analysis of the density of the fillers and the injection-molded samples, it was possible to determine the volumetric fraction of the filler in the composites and their porosity following Equation (3). Since the CS and BP fillers had a comparable density, it can be seen that the volumetric fraction of the filler is similar to those composite series. The higher volumetric content of the filler for the series containing VMs is due to the lower density and the extensive surface of expanded vermiculite, which was not entirely mechanically degraded during mechanical grinding and melt processing. According to the classification presented in [33], all samples reveal a low porosity, which excludes its strong effect on the mechanical properties of the samples and makes manufactured parts of good quality.

**Figure 4:**
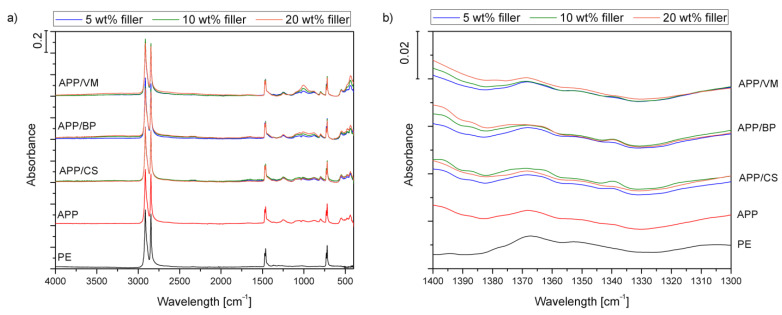
FTIR spectra of PE and PE-based composites in the range of 4000–400 cm^−1^ (**a**) and 1400–1300 cm^−1^ (**b**).

Figure 4a,b presents the spectra of the unmodified polyethylene matrix and composites containing applied fillers. Spectra of unfilled PE show an appearance typical for polyolefins [34]. The most significant absorption bands were noted around 2847 and 2914 cm^−1^ and were associated with the symmetric and asymmetric stretching vibrations of carbon–hydrogen bonds in the backbone of polyethylene. Signals attributed to these bonds’ bending and rocking vibrations were also noted at 1460, 1470, 718, and 729 cm^−1^. The positions of these signals are in line with the literature data on polyethylene materials [35]. A more detailed analysis of PE spectra confirms its type—high-density polyethylene (HDPE). According to Jung et al. [36], magnification of the 1300–1400 cm^−1^ region may provide essential insights related to the exact type of PE. Figure 4b (zoom 1300–1400 cm^−1^) points to the absence of the 1377 cm^−1^ absorption band and visible signals at 1367 and 1352 cm^−1^ that indicate HDPE. The filler incorporation hardly affected the position and magnitude of bands characteristic of HDPE, which points to the lack of matrix decomposition despite the use of hard and rigid mineral fillers. 

Spectra of the prepared composites show additional absorption bands related to the composition of the applied fillers. All spectra contain a minor peak around 1256 cm^−1^, characteristic of the stretching vibrations of P=O bands present in polyphosphate structures [37]. Bands related to the vibrations of single phosphorous–oxygen bonds were also noted around 800 and 880 cm^−1^ [38]. Moreover, materials containing basalt and vermiculite fillers show small signals in the range 990–1010 cm^−1^, typical for stretching Si–O bonds, as reported in previous work [39]. Bending vibrations of these bands were expressed by the signals around 450 cm^−1^. They were more pronounced for the composites filled with vermiculite, which is in line with our previous studies, indicating a powerful absorption band around 1000 cm^−1^ [26]. 

### 3.3. Thermal Properties

Thermal diffusivity (*D*) quantifies materials’ ability to conduct heat relative to their ability to store heat. This parameter may be determined using the Angström method, which is a steady-state measurement using an alternating-current heating plate [40]. This method in various modifications has been successfully used in multiple studies of polymers and their composites [28,29,41,42,43]. As previously discussed by Wenelska et al. [44], the determination of this property can help compare the thermal properties of PE-based composites and their flammability. Results obtained for considered materials showed a reciprocal tendency to those discussed in earlier studies because the presence of flame retardant and fillers lowered *D* values in comparison to unmodified polyethylene.

**Figure 5:**
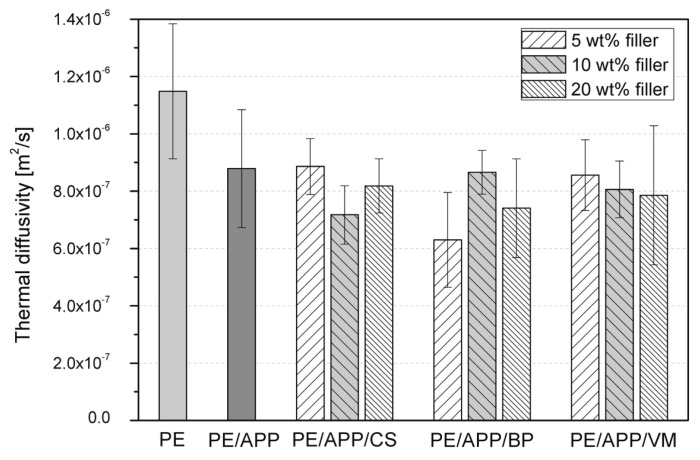
Thermal diffusivity of PE and PE-based composites.

Moreover, observed results showed that the overall change in thermal diffusivity, considering standard deviations, caused by the additional incorporation of inorganic fillers may be omitted. Figure 5 shows the averaged thermal diffusivity values obtained for PE and its composites. According to indirect density-based porosity measurements and SEM observations, the lowered thermal diffusivity may be connected with a high amount of well-dispersed additives and fillers, as well as the microporosity occurring in the composite structure. As discussed by Prociak et al. [29], the cell size, in the case of porous materials, may significantly influence the *D* value; even the smallest amount of pores may affect the thermal behavior of the polymeric materials. Simultaneously, it should be underlined that the measured thermal diffusivities for all materials are at a comparable level. Despite the highest porosity of the samples containing CS, the thermal diffusivity of the prepared composites did not differentiate itself from the other samples. The lowest *D* values noted for BP-filled composites should be connected with the better conductivity of basalt powder itself in comparison to VM and CS rather than to structural changes, including the presence of the voids in the injection-molded samples. 

**Figure 6:**
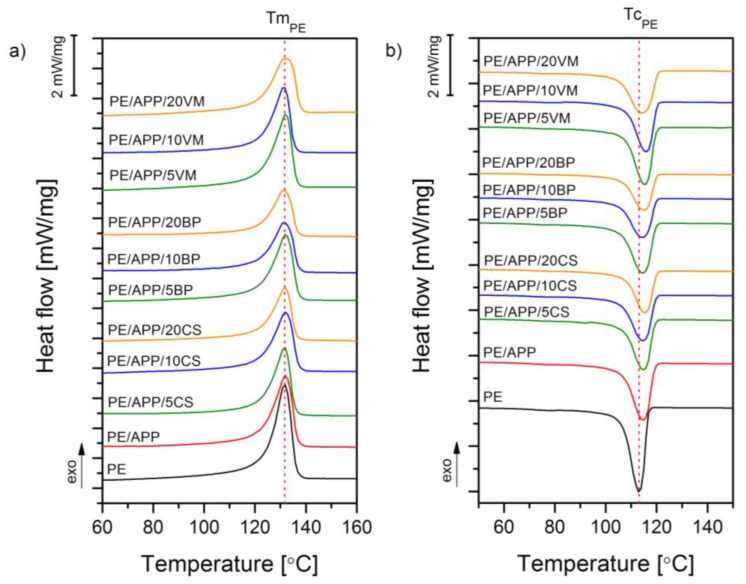
DSC curves obtained during the second heating (**a**) and first cooling (**b**).

The structure-related evaluation was based on a thermal analysis assessed by employing differential scanning calorimetry. Figure 6 shows the DSC curves from signals recorded during the second heating and first cooling. Additional thermal parameters such as the crystallization temperature (*T_C_*), second melting temperature (*T_M_*_2_), heat of fusion (Δ*H_M_*), and crystallinity level, calculated according to Equation (1), are collectively presented in Table 2. The nucleation density and size of the spherulites depend on the crystallization temperature, degree of undercooling, and molecular weight of the polymeric matrix [45]. Therefore, the incorporation of fillers may cause changes in the crystallization behavior by the heterogeneous nucleation and change the thermal diffusivity of the polymeric melt. Changes in nucleation are related to promoting spherulite generation on the filler particles’ surfaces, decreasing crystallites’ thickness, and causing the epitaxial growth of the spherulites. Considering the DSC method’s sensitivity, the observed melting and crystallization temperature changes of all APP-modified and inorganic filler composites can be negligible. At the same time, evident differences between individual material series are visible based on changes in the heat of fusion measured during the second heating process and the crystallinity calculated on its basis. Considering the low susceptibility of polyethylene to heterogeneous nucleation, the observed increase in crystallinity in the case of a 20 wt% addition of APP may be considered as having a substantial effect on the change in the PE structure. It should be mentioned that the achieved results are contrary to former studies [45], where incorporating APP into the HDPE matrix resulted in almost no effect on composite crystallinity. The difference may result from the different molecular weights of HDPE grades, which affect susceptibility to heterogeneous nucleation [46].

Interestingly, the addition of the lowest amounts of the inorganic fillers (VM and BP) resulted in the intensification of the nucleation effect, leading to an improved crystallinity level for the composites. However, for VM-filled composites, the crystallinity has been increasing gradually with the filler content; for basalt-filled composites, the opposite effect was noted. It should be mentioned that both micrometric and nanosized vermiculite were previously described as fillers with a confirmed nucleating ability on HDPE [45]; the exfoliated silicate layers may act as nucleation sites for the secondary nucleus of the composites during crystallization.

### 3.4. Thermal Stability and Fire Behavior under Forced-Flaming Conditions

**Figure 7:**
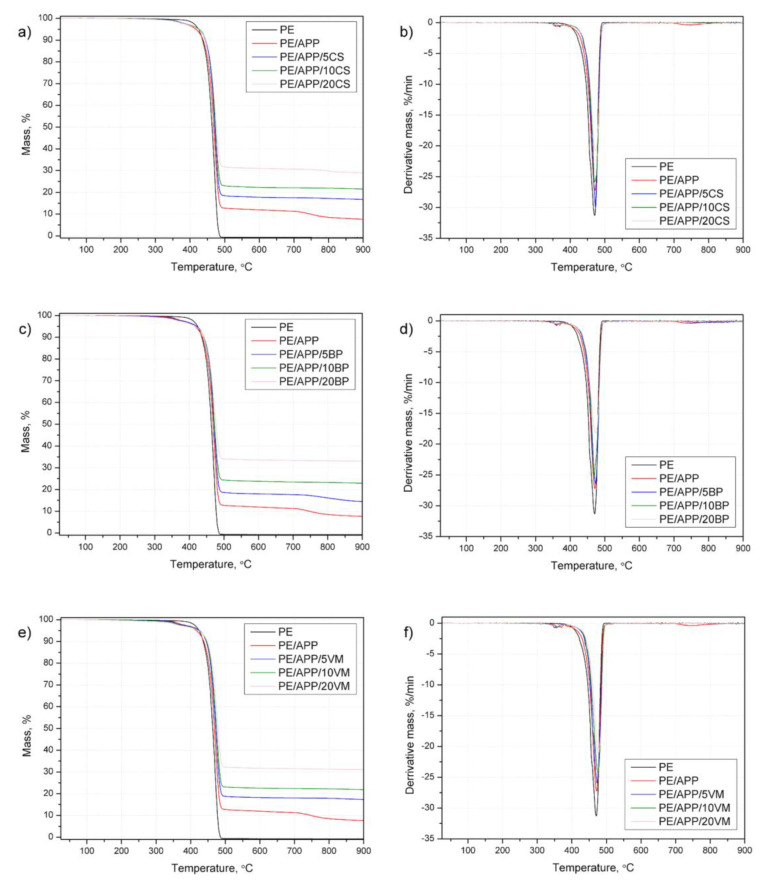
TG (**a**,**c**,**e**) and DTG (**b**,**d**,**f**) curves of PE and PE-based composites tested in a nitrogen atmosphere.

Results obtained with thermogravimetric analysis are summarized in Table 3 and Figure 7. The presented data highlight that incorporated flame retardants and inorganic fillers influence the thermal stability of the polymer. Unmodified HDPE degraded completely at approx. 500 °C. The most noticeable difference between polyethylene and PE/APP with fillers is that the polymer decomposed in a single step, whereas the composites present a two-step degradation. From Figure 7, it can be seen that APP/CS, APP/BP, and APP/VM have a similar course of DTG curves and show the primary weight loss at 348–376 °C and 469–477 °C. In the first one, the main products were H_2_O and NH_3_, resulting from the thermal decomposition of polyphosphate. In turn, the second was related, apart from the decomposition of HDPE, to the release of phosphoric, polyphosphoric, and metaphosphoric acids from APP [47,48,49].

The second stage of decomposition was delayed compared to polyethylene, and the decomposition rate was much lower (maximal reduction by 42% for PE/APP/20BP). Inorganic carbonaceous residues remained after the major decomposition step, between 14.5 and 33.0 wt%. According to the TG result, the APP combined with BP, excluding the system with 5 wt% of inorganic filler, had more residue than the other systems under the same decomposition condition. In the case of APP and APP with the lowest amount of filler, the carbonaceous char was consumed in the subsequent minor decomposition step above 500 °C. The main mass loss stage and the residue it generates may influence the fire behavior of materials [47,50]. 

In turn, the T_5%_ weight loss temperature corresponding to the onset temperature of each flame-retarded HDPE occurred earlier than that of unmodified polymer. This is due to the relatively low temperatures, in which APP begins to decompose and forms phosphorus or phosphoric acid, promoting chain stripping, cross-linking, and char formation [51]. The TGA results indicated that the developed systems might have potential as a flame retardant for PE.

**Figure 8:**
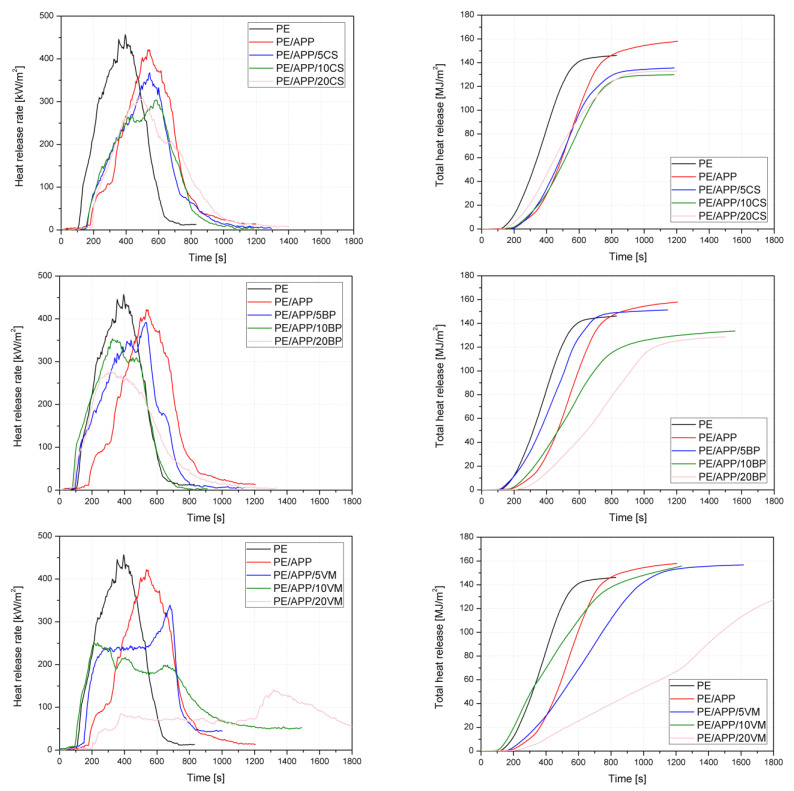
Heat release rate and total heat release curves of PE and PE-based composites.

The cone calorimeter provides parameters such as time to ignition (TTI), heat release rate (HRR), including the peak heat release rate (pHRR) and total heat release (THR), effective heat of combustion (EHC), maximum average rate of heat emission (MARHE), and specific extinction area (SEA), as shown in Table 3. The HRR and THR curves for unmodified HDPE and its composites are shown in Figure 8. 

Polyethylene burned intensively after ignition in 128 s with a pHRR of 414 kW/m^2^. The HRR curve course of HDPE is characteristic of a non-charring material, dominated by a pronounced pHRR. The addition of commercial fire retardant elongated the TTI without significant changes in the pHRR. Along with incorporating APP and CS, BP, or VM, in most cases, the time to ignition and burning time was prolonged, while the pHRR values decreased. Adding 5 wt% of inorganic components and 20 wt% of APP to HDPE resulted in a pHRR reduction from 6% (PE/APP/5BP) to 27% (PE/APP/5VM), whereas the samples with the highest additive amount showed reductions from 25% (PE/APP/20CS) to as high as 60% (PE/APP/20VM). The APP/VM was the most effective in reducing the burning intensity, and its use had changed the HRR curve’s course to the type of a charring or residue-forming polymer [47,52]. VM is known for its flame-retardant effects [18,53,54]. The decrease in HRR values also reduced indices illustrating the flame spread or fire growth rates, such as MARHE and FIGRA. The reduction in FIGRA was variable, whereas the decrease in MARHE, excluding HDPE modified with CS, showed gradual decline dependence according to the increasing number of fillers. The highest reduction in FIGRA and MARHE, 2.5 and more than 3 times, respectively, was noted for PE/APP/20VM. 

**Figure 9:**
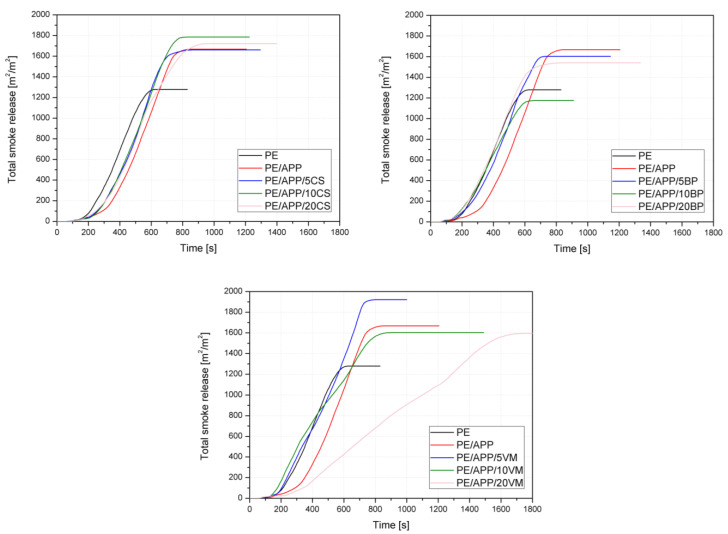
Total smoke release curves of PE and PE-based composites.

THR is a measure of the fire load, indicating incomplete combustion by reducing combustion efficiency and/or char creation [55]. APP and APP combined with 5, or in some cases 10 wt% inorganic components caused an increase in the total heat release (Table 4). From Figure 9, showing the total heat output versus time, it can be observed that HDPE modified with the investigated systems in most cases did not reach higher values than polyethylene during the first 800 s; however, the materials burned much longer. Samples with CS and 20 wt% VM did not achieve THR values as high as HDPE throughout the test, while the rest reached it just at the end of the flame burning. The highest reduction, equal to approx. 15%, was noted for PE/APP/20BP and PE/APP/10CS. In turn, the highest THR and the standard deviation were obtained for composites with VM. EHC of unmodified polyethylene is relatively high and similar to non-flame-retarded polyolefins [47,56]. The additions led to the change in the gas-phase activity, and excluding the samples from 10 and 20 wt% of VM, the decrease in EHC was observed. This indicates that fuel dilution effects due to the release of incombustible products, or flame inhibition due to the release of phosphorus species acting as radical scavengers, may have occurred [57]. Replacing some amount of the HDPE with inorganic components cannot be excluded, reducing the emission of volatile decomposition products into the combustion zone. The increased flame retardancy was accompanied by a moderate increase in the CO yield of between 1% and 29%. Moreover, the average yield of residue after the burning of 20APP was 18% and increased to 33–36% with increases in the content of the inorganic component. The change tendency of the residue yield in the CC test is similar to that in the TG analysis.

The total smoke release from the forced flaming combustion represents the cumulative smoke amount generated per unit area of the tested material [58,59]. The addition of a commercial flame retardant led to a considerable increase in TSR from 1211 m^2^/m^2^ to 1606 m^2^/m^2^. From the curves’ profiles in Figure 9, it is observed that most of the samples showed an increase in values over unmodified HDPE after 600 s of the test. The exception is PE/APP/20VM, which burned for more than 1800 s, and values higher than polyethylene appeared only for tests of about 1300 s. Notably, the relation between the data and the number of additives can be observed only for series with VM. A decrease in value with an increase in vermiculite content may be due to the release of a higher amount of water. Similarly, a specific extinction area, which corresponds to the surface of light-absorbing particles present in the smoke generated from 1 kg of material, increased due to the addition of developed systems. SEA values ranged from 373 to 521 m^2^/kg and were independent of the amount or even type of additives.

**Figure 10:**
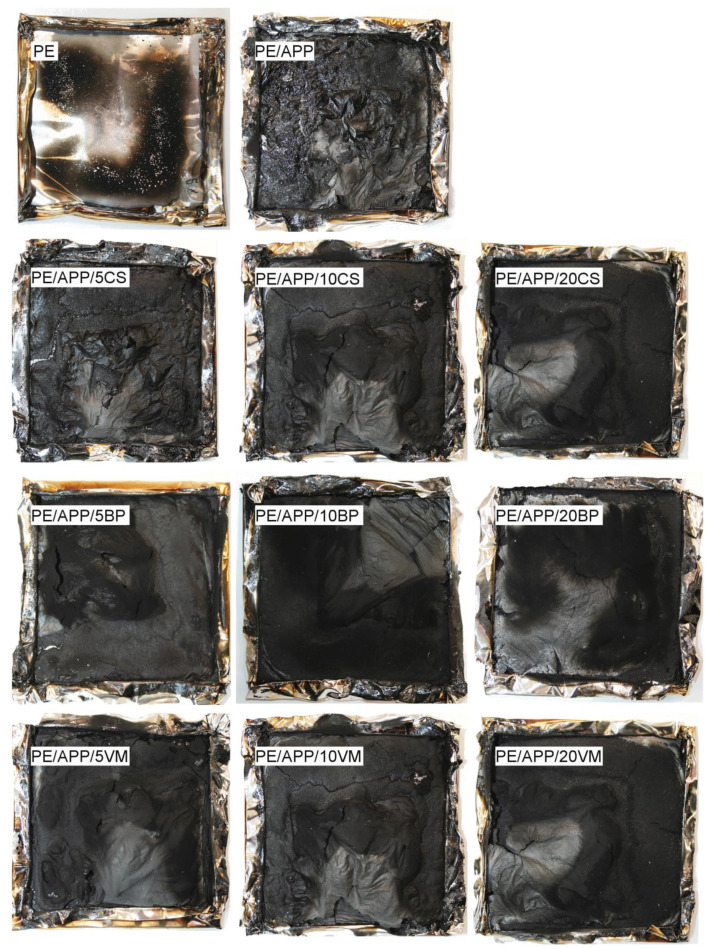
Sample appearance after the cone calorimetry test.

An effective protective layer suppresses the release of combustible volatiles as well as heat transfer into the materials, leading to a reduced HRR and prolonged burning time [47]. The residues of HDPE, polymer-modified with APP, and systems with BP, CS, or VM after cone calorimetric tests are shown in Figure 10. Unlike the composites, in the case of PE and PE/APP, there is no residue or only a little left. For the flame-retarded polyethylene, a continuous black char layer was formed on top of the burning materials. The increase in thickness was limited so that intumescence was practically ruled out. However, the formed residual layer was limiting heat transfer to the pyrolysis front and mass to the flame.

The change in the course of the curve with the reduction in pHRR and the considerable growth in residue exhibited a flame-retardant mode of action in the condensed phase. The residue yielded up to 0.3, demonstrating the formation of an inorganic carbonaceous char accompanied by an appropriate decrease in the fuel involved. In turn, the decrease in EHC and the increased CO yield and smoke production may indicate flame retardant effects in the gas phase. The increase in smoke emission and an EHC decrease by approx. 10% may suggest flame inhibition. Both flame retardant effects, the increase in residue due to charring and the decrease in EHC due to fuel dilution/flame inhibition, were detected. However, due to the linear increase in residue, charring started to outperform the gas-phase mechanisms with a high amount of additives [47].

### 3.5. Mechanical and Thermomechanical Properties

Table 5 summarizes the results of the mechanical and thermomechanical tests. The evaluation of the mechanical performance of polyethylene and its composites took into account the tensile test, Charpy impact strength, and hardness. The introduction of APP and inorganic fillers increased the elasticity modulus of the specimens. The addition of a flame retardant resulted in an increase in the Young modulus by 28%. In contrast, stiff domains of powder fillers caused a further improvement in this mechanical parameter. The PE/APP/20VM sample reveals the highest stiffness, which showed more than a two-fold increase in the stiffness compared to the reference material (unmodified PE). The increase in Young’s modulus caused by APP and fillers was reported earlier in the literature and is an expected effect resulting from stiff structures that block macromolecular mobility in traces of deformation [60,61,62]. At the same time, the most beneficial impact of the increase in stiffness caused by the introduction of VM may result from the increase in the degree of crystallinity for the polymer matrix, which usually leads to improvement of this mechanical parameter [63]. On the other hand, as was observed in SEM images, vermiculite-based composites, due to their complex structure, in the form of ground well-dispersed small plates as well as multilayered non-intercalated packets, reveal much higher volumetric content of the filler than CS- and BP-filled composite series, which also affect the composite stiffness [60,64]. Therefore, it can be supposed that while the final improvement of the elasticity modulus is caused by the presence of particulate fillers (CS and BP) acting as rigid stiff domains dispersed in the PE matrix, similar E values of VM-filled composites result from the higher crystallinity and volumetric content of the plate-shaped filler. 

The tensile strength of all the materials containing flame retardant and fillers decreased compared to the reference sample. However, it should be noted that even in the case of composites with the highest concentration, which contained a total amount of additives of 40 wt% (20 wt% APP and 20 wt% of filler), the reduction in the tensile strength was not so significant that it could constitute a considerable limitation in its use. The lowest *σ_M_* value (18.4 MP) was recorded for the PE/APP/20BP series, as it only has a deterioration of 20% compared to the reference sample. The particles of any of the additives used (FR and fillers) did not have a large shape factor to constitute a filler, enabling effective stress transfer and resulting in reinforcement for the polymer. Their presence led to the creation of points of stress accumulation during strain, causing the destruction of materials at lower strength values. It should be emphasized that the obtained tensile strength results are favorable, taking into account the high filling melt with inorganic materials introduced into the non-polar polymer without the use of a compatibilizer and surface modification, which, according to previously published studies [64], are crucial from the point of view of obtaining the mechanical properties of particulate-shaped composites.

A phenomenon of decreasing elongation at the break of polymers due to the addition of fillers and modifiers is widely described and reported in the literature [65,66,67] and is connected with the accumulation of stresses at the polymer-filler interphase. According to Pukanszky et al. [68], the dominant effect affecting changes in the mechanisms of micromechanical deformations is debonding, understood as the loss of adhesion between the polymer matrix and the filler. Considering the lack of additional compatibilizers enhancing interfacial adhesion, the dominant factor influencing the limitations of elongation at reaction is not so much the particles’ size and shape as it is the filler’s volumetric content [68,69]. The minimum modifier content (APP) in the considered case was 20 wt%. The composites were made by adding 5 to 20 wt% of the inorganic filler. All materials showed a drastic drop in elongation at break compared to unmodified PE. It should be emphasized that the individual composite series showed different *ε_b_* values at a comparable mass concentration of the filler, which resulted from the different volumetric content and the degree of dispersion in the matrix. However, these values, from the point of view of the functional properties of the final products, can be considered comparable. 

All the impact tests of polyethylene and its composites were performed on the notched specimens; therefore, all of them were fully broken. The addition of powder fillers and a flame retardant reduced the impact resistance. Interestingly, incorporating an inorganic filler to the polyethylene modified with APP did not cause any additional reduction in the impact strength of the composite compared to the PE/APP series. The PE/APP/20CS and PE/APP/5BP samples were characterized by their higher impact strength. The first series mentioned above had the highest impact strength among the modified material series. The most important, that is, more than threefold, deterioration of the impact toughness was noted for composites modified with vermiculite. The impact strength of composites reinforced with dispersion fillers decreases with the increasing volume of the filler [70,71]. VM appeared both in the form of single dispersed plates in the polymer matrix and exfoliated flakes with concertina-shaped domains. Composites manufactured with their use were characterized by a more significant volumetric share of filler in the matrix than other series and an increased concentration of stresses around the unsaturated spaces of the exfoliated filler not intercalated by the polymer. The decrease in the impact strength of the thermoplastic composites is often referred to as the increase in the composite stiffness caused by rigid filler structures dispersed in a polymeric matrix. At the same time, the stress concentration occurring around particles may result in the appearance of additional crack propagation points during the dynamic loading of the material [61,71]. Paradoxically, as demonstrated by Sewda and Maiti [71], the use of a compatibilizer to increase adhesion between the filler and the polymer may reduce the normalized relative impact strength value. Thus, the noticeably greater brittleness of the VM-filled composites observed in the case under consideration should be assigned with a different adhesion to the polyethylene matrix but with an increased volumetric fraction of the plate shape of the filler. Moreover, the drastically smaller size of the filler and the resulting shortened interparticle distance in the case of VM-filled composites also plays a role in worsening the response of the composites manufactured with their share to the impact load [70]. To summarize, compared to the lowered elongation at break of the modified polyethylene series, the materials should be classified as brittle.

The presence of fossil or waste-originated inorganic fillers, characterized by an increased hardness compared to the polymer matrix, leads to an increase in the parameter hardness of composites produced with their use [35,62,69,72]. The incorporation of APP improved the Shore D hardness by 5%, while the additional introduction of inorganic fillers affects a further gradual increase in this mechanical property, along with a rising filler content. The highest hardness was shown for the PE/APP/20VM series samples. However, it should be emphasized that the differences in hardness between the composite batches made using different fillers negligible. It is justified considering both the similar chemical composition and the dominant share of SiO_2_ in the fillers [14,26,35]. 

Table 5 also summarizes the results of thermomechanical tests determined in static point load (*VST*) and three-point bending (*HDT*) conditions. By analyzing the thermomechanical properties of the composites, it can be concluded that introducing all types of inorganic fillers increased their thermomechanical stability in the case of the highest concentration of fillers in the composite. The modification effectiveness of individual fillers was varied. Interestingly, for all fillers, the increased hardness values were comparable, so it can be concluded that the changes in *VST* were additionally associated with changes in the macromolecular structure of the polymer matrix. At the same time, taking into account the results of DSC studies, changes in the thermomechanical properties of certain static conditions can only be associated with an increased degree of crystallinity in the case of a series of materials reinforced with vermiculite. In the case of the remaining series, there was no significant increase in crystallinity caused by the fillers and APP. Therefore, in the case of CS- and BP-filled composites, the dominant role in increasing the load-carrying capacity and increased resistance to point loading was played by the rigid filler domains in the polymer matrix.

In contrast, in the case of VM-containing composites, the effectiveness of the interaction was additionally increased by modifying the crystal structure of the polymer. It should also be noted that for PE/APP/VM composites, higher *VST* values than *HDT* were recorded in all cases, which may be related to the plate-shaped structure of the filler, which creates targeted structures in the wall layers of the injected samples during the technological process, resulting in an increased resistance to operation of the indenter during mechanical (hardness tests) and thermomechanical (*VST*) measurements. Referring to the research presented by Rusu et al. [73], exceeding the concentration of the filler accompanying the appearance of agglomerated zinc powder filler structures in HDPE resulted in the lack of dependence of the influence of the increasing amount of filler on the flexural modulus, although it did not reduce the effectiveness of the beneficial effect on hardness and *VST*. Given the SEM observations of PE-based composites and the noticed differences in the distribution and particle size of the filler, the lack of an exact correlation between *VST* and *HDT* is justified.

## 4. Conclusions

Correlative analysis of polymer composites produced based on ammonium-polyphosphate-modified polyethylene using three types of thermally stable inorganic dispersion fillers has been realized. According to the assumptions, adding fillers increased the stiffness and hardness of composites both at room and at elevated temperatures, which may significantly increase the applicability range of the flame retardant polyethylene. Realized studies using a cone calorimeter showed that adding a micrometric filler with high thermal stability causes some beneficial effects in terms of reducing flammability. Introducing a filler with a complex plate shape (ground expandable vermiculite) allowed for the obtainment of a synergistic effect, significantly reducing the heat rate release. This may be related to the presence of a fine fraction of the filler in the form of plates and the presence of unbroken packets of exfoliated filler without interactions with polymer. Their presence in the swelled surface of APP-modified polymer during combustion caused the effect of the formation of a ceramic surface layer and increased barrier properties.

The introduction of all types of fillers resulted in the deterioration of the overall mechanical performance of the composites compared to polyethylene. However, it should be added that the changes in the tensile strength and impact strength were not at the level that would significantly limit the use of developed compositions.

## Figures and Tables

**Table 1:** Physical parameters of injection-molded PE and PE–composite samples.

Sample	Density	Volumetric Content of the Filler	Porosity
	[g/cm^3^]	[%]	[%]
PE	0.949 ± 0.003	-	-
PE/APP	1.043 ± 0.001	10.95	1.33
PE/APP/5CS	1.083 ± 0.001	13.2	1.45
PE/APP/10CS	1.131 ± 0.001	15.6	1.09
PE/APP/20CS	1.219 ± 0.001	21.2	2.27
PE/APP/5BP	1.081 ± 0.001	13.2	1.59
PE/APP/10BP	1.139 ± 0.002	15.7	0.38
PE/APP/20BP	1.239 ± 0.001	21.4	0.48
PE/APP/5VM	1.087 ± 0.001	13.4	0.91
PE/APP/10VM	1.129 ± 0.001	16.0	1.04
PE/APP/20VM	1.222 ± 0.001	22.0	1.44

**Table 2:** Thermal parameter obtained from DSC experiments.

Sample	*T_C_*	*T_M_*_2_	*ΔHm*	*Xc*
	[°C]		[J/g]	[%]
PE	113.1	131.7	198.2	68.8
PE/APP	114.7	131.9	162.7	70.6
PE/APP/5CS	114.8	131.6	141.7	65.6
PE/APP/10CS	114.6	131.9	140.6	69.7
PE/APP/20CS	115.4	131.7	118.4	68.5
PE/APP/5BP	114.5	132.0	155.1	71.8
PE/APP/10BP	114.3	131.4	126.1	62.5
PE/APP/20BP	115.0	131.2	110.5	63.9
PE/APP/5VM	115.3	132.2	162.5	75.2
PE/APP/10VM	115.6	131.7	155.5	77.1
PE/APP/20VM	114.2	132.1	148.0	85.6

**Table 3:** Thermal properties of PE and PE-based composites tested in a nitrogen atmosphere.

Sample	T_5%_	1st DTG Peak	2nd DTG Peak	Residual Mass at 900°C
	[°C]	[°C; %/min]		[%]
PE	424	-	471; −31.30	0
PE/APP	417	370; −0.71	404; −27.90	7.6
PE/APP/5CS	423	371; −0.59	473; −30.00	16.7
PE/APP/10CS	426	376; −0.50	470; −25.96	21.5
PE/APP/20CS	423	374; −0.45	474; −20.82	28.9
PE/APP/5BP	422	361; −0.74	473; −23.39	14.5
PE/APP/10BP	418	361; −0.51	469; −25.40	22.9
PE/APP/20BP	418	363; −0.51	474; −18.24	33.0
PE/APP/5VM	425	355; −0.72	473;−25.96	17.4
PE/APP/10VM	423	362; −0.61	476; −24.32	21.9
PE/APP/20VM	422	348; −0.48	477; −21.07	31.1

**Table 4:** Cone calorimeter data of PE and PE modified with fire-retardant systems.

Materials	TTI, s	pHRR, kW/m^2^	MARHE, kW/m^2^	FIGRA, kW/m^2^	THR, MJ/m^2^	EHC, MJ/kg	Residue, %	CO Yield, kg/kg	SEA, m^2^/kg
PE	128 (10)	414 (37)	231 (15)	1.0 (0.1)	148 (5)	42 (2)	9 (0)	0.0249 (0.0)	332 (14)
PE/APP	174 (8)	423 (35)	195 (4)	0.8 (0.0)	153 (12)	41 (2)	18 (1)	0.0295 (0.0)	411 (25)
PE/APP/5BP	123 (20)	391 (5)	210 (15)	0.7 (0.1)	154 (15)	41 (2)	18 (3)	0.0281 (0.01)	456 (40)
PE/APP/10BP	119 (52)	327 (58)	203 (51)	0.9 (0.4)	137 (7)	39 (2)	23 (5)	0.0272 (0.0)	373 (62)
PE/APP/20BP	131 (67)	277 (71)	166 (47)	0.7 (0.4)	128 (3)	38 (1)	33 (5)	0.0260 (0.0)	419 (82)
PE/APP/5CS	159 (27)	366 (16)	176 (7)	0.7 (0.1)	135 (5)	39 (2)	23 (2)	0.0314 (0.0)	469 (10)
PE/APP/10CS	154 (19)	286 (26)	162 (3)	0.5 (0.0)	127 (7)	38 (1)	26 (0)	0.0321 (0.0)	491 (25)
PE/APP/20CS	165 (55)	310 (34)	166 (13)	0.8 (0.2)	132 (12)	38 (2)	33 (1)	0.0320 (0.0)	479 (27)
PE/APP/5VM	158 (13)	301 (69)	162 (23)	0.4 (0.1)	156 (3)	41 (1)	20 (0)	0.0311 (0.)	521 (33)
PE/APP/10VM	114 (32)	252 (47)	157 (33)	0.8 (0.5)	158 (19)	44 (5)	36 (2)	0.0310 (0.0)	404 (47)
PE/APP/20VM	185 (84)	165 (40)	93 (42)	0.3 (0.4)	140 (23)	43 (5)	25 (1)	0.0251 (0.0)	422 (138)

The values in parentheses are the standard deviations.

**Table 5:** Mechanical and thermomechanical properties of PE and PE-based composites.

Sample	Tensile Strength, *σ_M_*	Elasticity Modulus, *E*	Elongation at Break, *ε*	Charpy Impact Strength, *a_k_*	Shore D Hardness	Vicat Softening Temperature, *VST*	Heat Deflection Temperature, *HDT*
	[MPa]		[%]	[kJ/m^2^]	[°ShD]	[°C]	
PE	23.0 (1.4)	673 (32.5)	98 (12)	3.64 (0.38)	61.2 (0.6)	74.5 (0.8)	61.9 (4.1)
PE/APP	19.9 (0.66)	862 (13.9)	12 (3.2)	1.84 (0.15)	64.3 (1.0)	73.6 (0.3)	73.0 (4.5)
PE/APP/5CS	19.4 (0.24)	924 (39.8)	7.7 (0.96)	1.79 (0.14)	65.4 (0.6)	73.5 (1.0)	63.2 (0.5)
PE/APP/10CS	20.6 (1.75)	1260 (100)	5.3 (1.80)	1.20 (0.31)	64.8 (0.8)	76.0 (0.6)	73.6 (6.0)
PE/APP/20CS	19.9 (0.65)	1335 (49.5)	3.7 (0.08)	2.22 (0.09)	66.1 (0.5)	77.6 (0.4)	80.4 (6.4)
PE/APP/5BP	19.7 (0.66)	978 (48.8)	9.3 (0.87)	2.05 (0.67)	65.1 (0.8)	73.5 (1.0)	73.3 (0.3)
PE/APP/10BP	18.6 (0.43)	1070 (55.0)	8.6 (0.43)	1.62 (0.62)	66.1 (0.8)	74.8 (1.3)	77.9 (5.6)
PE/APP/20BP	18.4 (0.31)	1200 (102)	2.1 (0.09)	1.04 (0.22)	66.5 (1.6)	81.6 (0.9)	106.5 (5.4)
PE/APP/5VM	19.3 (0.53)	976 (42.1)	6.3 (1.1)	1.43 (0.25)	65.2 (0.7)	75.6 (0.4)	74.0 (1.2)
PE/APP/10VM	19.1 (0.45)	1070 (61.1)	4.7 (0.34)	1.21 (0.06)	66.4 (0.8)	77.9 (0.9)	77.7 (5.2)
PE/APP/20VM	19.0 (1.37)	1450 (115)	1.9 (0.32)	1.00 (0.03)	67.9 (1.2)	82.3 (1.0)	79.4 (2.3)

## Data Availability

Not applicable.
